# Developing numeracy skills using interactive technology in a play-based learning environment

**DOI:** 10.1186/s40594-018-0135-2

**Published:** 2018-10-11

**Authors:** Tess Miller

**Affiliations:** University of Prince Edward Island, 550 University Avenue, Charlottetown, PE C1A 3C2 Canada

**Keywords:** Interactive technology, iPads, Early learning, Mathematics, Mobile learning device play-based learning, Assessment

## Abstract

**Background:**

The purpose of this study was to measure the impact of interactive technology in the form of mathematical applications (apps) delivered using iPads on kindergarten children’s learning of number sense in a play-based learning environment. Secondly, factors influencing the use of interactive technology in a play-based environment were examined. This technology was introduced to a small (*n* = 13) rural kindergarten classroom using an experimental design embedded in a mixed methods approach.

**Results:**

The teacher was keen to introduce technology to her class but was self-described as a beginner in using iPads for personal or teaching tasks. Small gains were noted between the control and intervention groups but they were not significant. Further, children were observed collaborating which supported prior research. Another observation was related to attention span, when an app became too challenging children would abandon the app or use a trial and error method to move to the next level. Lastly, when given choice, children were drawn to creative and entertaining apps rather than apps that were more pedagogically accurate but less creative. Although there was not a large gain in achievement, using interactive technology promoted student collaboration and engagement in a play-based learning environment.

**Conclusions:**

Small gains in mathematics achievement and high levels of engagement suggest that using interactive technology in the kindergarten classroom enhances learning of mathematics. Factors influencing the use of interactive technology included the quality of the app such that creative and fun apps promoted children’s engagement in learning mathematics. The level of difficulty of an app was a second factor influencing children’s use of interactive technology. If the difficulty level was too challenging, children became disengaged with the app.

## Background

In the next era of technological advancements, we can only envision futuristic developments such as no-touch interfaces and sensors that read and predict our movements (Loganathan 2013). However, for the current generation, we are enjoying the developments in technology that have focused on interactive tablets among a host of other interactive gadgets currently on the market. The intuitive design of iPads positions them for use in educational settings, including early years classrooms (Warmoth 2013). Their design was built on mental models of how we perceive an experience (Weinschenk 2011). For example, when reading a book, we turn pages by using the index finger to flip to the next page. This method of turning pages has been modeled on the iPad where the user also uses his/her index finger to flip to the next page. Interacting with an iPad in this manner facilitates data flow through the interface (i.e., what the user sees on the screen) linking the user and technology and is referred to as interactive technology (Large 2016). Page flipping and other interactive features (e.g., voice recognition) on an iPad make using this form of technology simple or intuitive even for the youngest of learners.

The introduction of iPads into early learning classrooms has revealed gains in student achievement (Bebell et al. 2012); however, measuring gains in achievement should not be the sole factor determining the merit of interactive technology. Leveraging student engagement in a discipline like mathematics also makes iPads a worthy investment for the classroom given that they can be used to gather information, read a book, take photos, record physical activity, make artistic drawings, and learn about literacy or numeracy through the use of stimulating and creative applications (apps). Given the abundance of apps currently available, examples of how iPads can be used to engage children and promote learning is much larger than what is posited here. In the next era of educational technology, we need to think of the iPad as a manipulative that children can choose from a host of other manipulatives to discover new concepts.

The purpose of this study is to measure the impact of interactive technology in the form of mathematical applications delivered using iPads on kindergarten children’s learning of numeracy in a play-based learning environment (Fesseha and Pyle 2016). In particular, numeracy concepts were focused on the number-sense strand (Department of Education, Early Learning and Culture of PE 2008). Secondly, factors influencing the use of interactive technology in a play-based environment were examined. The research questions posed in this study are as follows: To what extent does the use of mathematical apps using iPads enhance children’s learning of numeracy in kindergarten? What factors influence children’s use of interactive technology in a play-based learning environment?

## Literature

Considering the relatively short evolution of iPads that were introduced in 2010 with Apple’s launch of iPad 1 (Ritchie 2014), a significant body of literature has been published on the use of iPads in education, building on literature reporting on the use of desktop computers in education. In a systematic review of what the authors described as mobile learning, Crompton et al. (2017) reviewed 113 studies of which four were at the pre- or kindergarten levels and these four studies were conducted in 2014 and 2015 suggesting interactive technology was slowly making its way into the early grades. Unfortunately, these authors did not cross list their review to identify the subject domains the four studies were conducted in.

Of the research focusing on technology in early learning, studies focused on children and teachers’ perceptions of technology (Knezek and Christensen 2002; Tsitouridou and Vryzas 2003) as well as the use of technology in literacy development (e.g., Chiong and Shuler 2010; Flewitt et al. 2015; Primavera et al. 2001). Fewer studies have been in the area of numeracy (Clements and Sarama 2007). This literature review begins with teachers’ perceptions towards using technology given that teachers’ comfort with technology, including the teacher in this study, has been shown to impact the use of technology in the classroom (Simon et al. 2013). The second part of this literature review focuses on literature exploring the effective use of iPads in the area of literacy and numeracy. This review concludes with a look at studies that advocated against the use of technology in early learning.

### Teachers’ perceptions towards using technology

For many preschool teachers, their use of technology is less than teachers in higher grades and was limited to downloading images for instructional purposes and digital cameras (Public Broadcasting Service and Grunwald Associates 2009, 2011). This absence of technological integration into early years classrooms is most likely due to limited opportunities for professional development on interactive technology as well as the lack of technology itself. Hence, to advance interactive technology in early years classrooms, it is important to recognize that teachers need professional development on the appropriate use of technology in the classroom (Simon et al. 2013), as well as opportunities to acquire technology. In a case study of four kindergarten teachers by Lu et al. (2017), teachers’ experiences using iPads in a literacy context was also found to be beneficial as it allowed teachers to meet the demands of creating individual assessments or work on lesson preparation as the iPads “functioned like an extra teaching assistant, providing feedback to students” (Lu et al. 2017, p.19).

### Literacy

Studies employing interactive technology in the form of tablets reported improved motivation, supported learning in small groups, and independent work as well as gains in vocabulary and phonological awareness (Dobler 2012; Hutchison et al. 2012; Flewitt et al. 2015; Hutchison and Reinking 2011; Simon et al. 2013; Takac et al. 2015). For example, Simon et al. (2013) concluded that tablets in addition to desktop computers supported learning in small groups or individually. These researchers also noted a tendency for longer periods of use especially when children had choice in their activity. Similarly, Flewitt et al. (2015) introduced iPads in early learning for the purpose of literacy development, which included writing and video recording of stories for sharing with the class. One iPad was given to a classroom with 3- and 4-year-olds and another iPad to a class of 4- and 5-year-olds for 2 months. Each iPad contained a story-creation app as well as a number of other learning apps. Training was provided to both instructors and data collection consisted of surveys, observations, and conversations. These researchers reported increased motivation and use of iPads. This was especially noted among children who were not easily engaged in traditional writing tasks. Further, the touch screen interface was reported to be easier than a keyboard. Similar to findings reported by Simon et al. (2013), the iPads fostered small group and independent learning. At the same time, children were observed helping each other use the iPads which is aligned with the findings from Shifflet et al. (2012) who reported that pre-school children using tablets developed a collaborative approach to learning which enhanced their social skills.

In terms of academic gains, an experimental study revealed that kindergarten children in both the control and experimental groups (i.e., with iPads) showed gains as measured using the Rigby Benchmark Assessment and the Children’s Progress Academic Assessment but the differences between groups were not statistically significant (Bebell et al. 2012). However, on a third measure, the Observation Survey of Early Literacy Achievement assessment, a statistically significant difference in phonemic awareness was reported where children in the iPad group scored higher (Bebell et al. 2012). Based on this study, the introduction of iPads in early learning did not appear to hinder learning, and in some areas of literacy, they enhanced children’s understanding of the discipline.

### Numeracy

Of the studies that examined early learning of mathematics using tablets, most studies reported gains in achievement or positive experiences (Alade et al. 2016; Dejonckheere et al. 2015; Hubber et al. 2016; Hung et al. 2015; Kosko and Ferdig 2016; Mattoon et al. 2015; Outhwaite et al. 2017; Presser et al. (2015); Reeves et al. 2017; Stubbe et al. 2016). However, Bebell and Pedulla’s (2015) longitudinal study examining the impact of mathematics apps on achievement in grades kindergarten (K) to 2 did not reveal any consistent gains. Despite different outcomes, each of these studies dispelled the argument that iPads and other digital manipulatives were not edutainment but rather effective learning aids (Baird and Henninger 2011).

A few studies narrowed their focus to a small number of apps or a specific mathematical skill, which provided insight on the connection between the app and student gains for young children. For example, Reeves et al. (2017) selected apps that focused on skills related to counting, sequencing, and early addition. In each area of skill development, gains were reported. Dejonckheere et al. (2015) also focused on a specific skill and reported a gain in achievement. These researchers used tablets to allow 4- to 6-year-olds to play on a digital number line exploring concepts related to estimation. This focus on one numeracy concept applying different strategies for estimation revealed a significant gain in accuracy of estimation for the two groups that were given strategies but not for the control group. Likewise, Presser et al. (2015) focused on skills related to subitizing (recognizing how many in a set without counting) and equi-partitioning (splitting an area or set into equal groups) using an app known as *Next Generation Preschool*. Gains in numeracy skills were also reported in their study.

Along the same focus, Kosko and Ferdig (2016) examined gains in achievement but approached their study from the perspective of selecting apps that were pedagogically accurate and aligned with curriculum. These researchers reported that well-designed mathematics apps improved achievement and concluded that well-designed mathematics apps can support student learning but more research was needed to explore the extent to which these apps improved learning. Given the number of mathematics apps available on the iPad, it is particularly important to consider characteristics of mathematics apps to better understand the impact, if any, an app has on student learning. Further, neither of these studies centered on a play-based learning environment that allowed for flexible and creative use of time and space or provided choice in selecting an app (Steglin 2005). Hence, the characteristics of apps that attracted children are largely unknown.

### Advocates against interactive technology

Although Dinehart (2015) was an advocate against the use of technology in early learning, citing that it diminished children’s fine motor skills, she did not consider apps designed for early childhood education that fostered fine motor skills through writing letters and numbers. Other concerns against the use of technology in early learning focused on the amount of time spent viewing screens. Vanderloo (2014) reported that children between 4 and 7 years of age spent an average 1.5 to 7.0 h viewing screens each day. Unfortunately, there was no differentiation between viewing screens for different activities such as watching a movie, watching a lesson on the interactive whiteboard, or playing games on an iPad (Vanderloo 2014). The sedentary nature of viewing screens was the catalyst for Vanderloo’s work, which is undoubtedly a concern; however, moderation of screen viewing may better guide the use of technology in early learning rather than banning it all together. This position is more aligned with the National Association for Education of Young Children (2012) who differentiated between screen viewing for interactive activities (e.g., mathematical apps) and non-interactive activities (e.g., movies) to promote active learning using technology.

When drawing on the literature presented above, it is reasonable to conclude that young children experienced gains when using iPads to learn about literacy and numeracy, and in one study, gains were long term (Outhwaite et al. 2017). When these gains were compared to a control group, the gains were not always statistically significant. Concerns related to excessive screen viewing were tempered by differentiating between interactive learning versus non-interactive learning. Since most studies narrowed their focus to a few mathematical apps, there is much to learn about the characteristics of apps that children gravitate towards when left to their own accord in a play-based learning environment.

## Theoretical framework

The Technological Pedagogical Content Knowledge (TPACK) framework was utilized to explore the implications of using mathematical apps installed on iPads in an early learning context. Integrating technology to enhance learning requires knowledge related to the subject content (i.e., numeracy), pedagogy, and technology (Mishra and Koehler 2006). Based on the TPACK framework, technology can be successfully integrated into learning when these three domains are successfully woven together. Hence, when apps were selected for this study on early numeracy concepts, the pedagogy had to be aligned with children’s level of cognitive development, and at the same time, the technology had to be simple and intuitive so that kindergarten children could be successful using it.

This research also builds on Naismith et al.’s (2004) theories in identifying six theory-based categories of activities for interactive technology: behaviorist, constructivist, situated, collaborative, internal and lifelong, and learning and teaching support. These researchers described behaviorist activities as those that primarily aim to change behavior through reinforcement of a stimulus such as feedback. For example, when a student responds correctly to a mathematics question, a pleasing sound or an animated character may appear in an app designed for kindergarten level students. In contrast, constructivist activities were described as activities that call on the student to apply what they know to new contexts and build new learning. The game, *Environmental Detectives*, was created for high school students who engage in the game as environmental engineers tasked with solving a problem is an example of a constructist activity/game. For a younger audience, *Minecraft* is similar in that students create and manipulate objects and engage in a task. When selecting apps for this study, there were no constructivist activities/games for the iPads that were aimed at the early learner.

In returning the focus to the theory-based categories, the remaining four theory-based categories of activities/games for interactive devices involved a higher cognitive engagement and advanced interactive software, which is appropriate for students in the senior grades. Subsequently, interactive activities designed for early learners appears to be founded on the behaviorist theory-based category which is similar to the finding in Bray and Tangney’s (2017) systematic review of literature focused on using technology in mathematics education. These researchers identified a wide range of technologies being used in different contexts within the middle and senior mathematics classrooms with a predominance of constructivist and social constructivist tasks. Such tasks appear to be aligned with higher grade levels where the curriculum calls for higher levels of inquiry-based, student-centered, and collaborative approaches to learning mathematics leaving the behaviorist types of activities/games for the younger students.

## Context

This study was implemented in a small rural Canadian primary school. The kindergarten teacher selected to be involved in the study was a veteran teacher with over 20 years of early years teaching experience. Although the teacher was not fluent with iPad technology, she was keen to learn and introduce the technology to her classroom. Funds to purchase four iPads with protective childproof cases and glass screen protectors, apps, stylus, and child safe headsets (control the volume such that the sound does not increase above 85 dB) were obtained through a small university grant. The agreement between the researcher and the primary school allowed the iPads and supporting technology to remain a property of the kindergarten class. Ethics permission was obtained through the university research ethics board as well as the local school board ethics authority.

## Methods

A mixed methods design was applied using qualitative and quantitative data to explore the impact of mathematical apps on the learning of numeracy skills and the factors influencing the use of this technology in an early years setting. The qualitative data included field notes documenting conversations during the training session with the kindergarten teacher as well as observation notes of students using iPads. The experimental component provided the quantitative data. In this aspect of the study, the quantitative measures were the pre- and post-test measures for the experimental and control groups. The rationale for choosing a mixed methods design was to provide a wider perspectives of the context of using interactive technology in an early years setting as well as to have greater understanding of the research questions posed in this study (Johnson and Onwuegbuzie 2004; Almalki 2016). A mixed methods approach allows the researcher to compensate for the fundamental weaknesses that are associated with using only a quantitative or qualitative study (Almalki 2016).

### Study design

The participating kindergarten teacher was selected for this study because she had demonstrated excellent knowledge about teaching mathematics to kindergarten children and was keen to engage in a research project. Prior to commencing the study, four iPads with several language arts and mathematics apps that were aligned with the curriculum were selected in collaboration with the teacher and researcher. The researcher and teacher met three times prior to commencing the study to select and experiment with the apps.

One week prior to commencing the study, one iPad was placed at each play station for approximately 20 min each day for 1 week. Children had the choice of using the iPads without receiving guided instructions. This pre-exposure to the iPads was intended to remove any novelty effects that might influence a gain in numeracy skills (Gravetter and Forzano 2011).

In the experimental phase, 13 children in the kindergarten class, aged four and five, were randomly selected to one of two groups. One group received a 2-week intervention involving the use of iPads to learn numeracy concepts each day and the other group followed the traditional play-based learning activities that focused on numeracy development, in particular, concepts of number sense. In a conversation with the teacher, she believed that students would be able to master the outcomes being learned in the 2-week period.

Children’s mathematical skills from both groups were measured at the beginning of the study (time 1) with 30 items that were aligned with the curriculum being taught and again following the 10-day intervention period (time 2). At the conclusion of the study, the control group was introduced to the iPads in the same manner as the experimental group (i.e., 10 days) for the purpose of ensuring equal opportunity to engage with the iPads.

The intervention consisted of using interactive technology in a play-based mathematics classroom in lieu of the teachers’ originally planned play-based lessons. A teacher-trained, research assistant removed Group 1 children to another classroom during the time designated for learning mathematics, which was approximately 20 min each day. This group of children was introduced to various mathematical apps while the control group, Group 2, children followed the play-based activities that fostered the same skill development that was in the apps. For example, one of the apps fostered the development of writing numerals and a play-based activity required students to trace numerals.

**Fig. 1:**
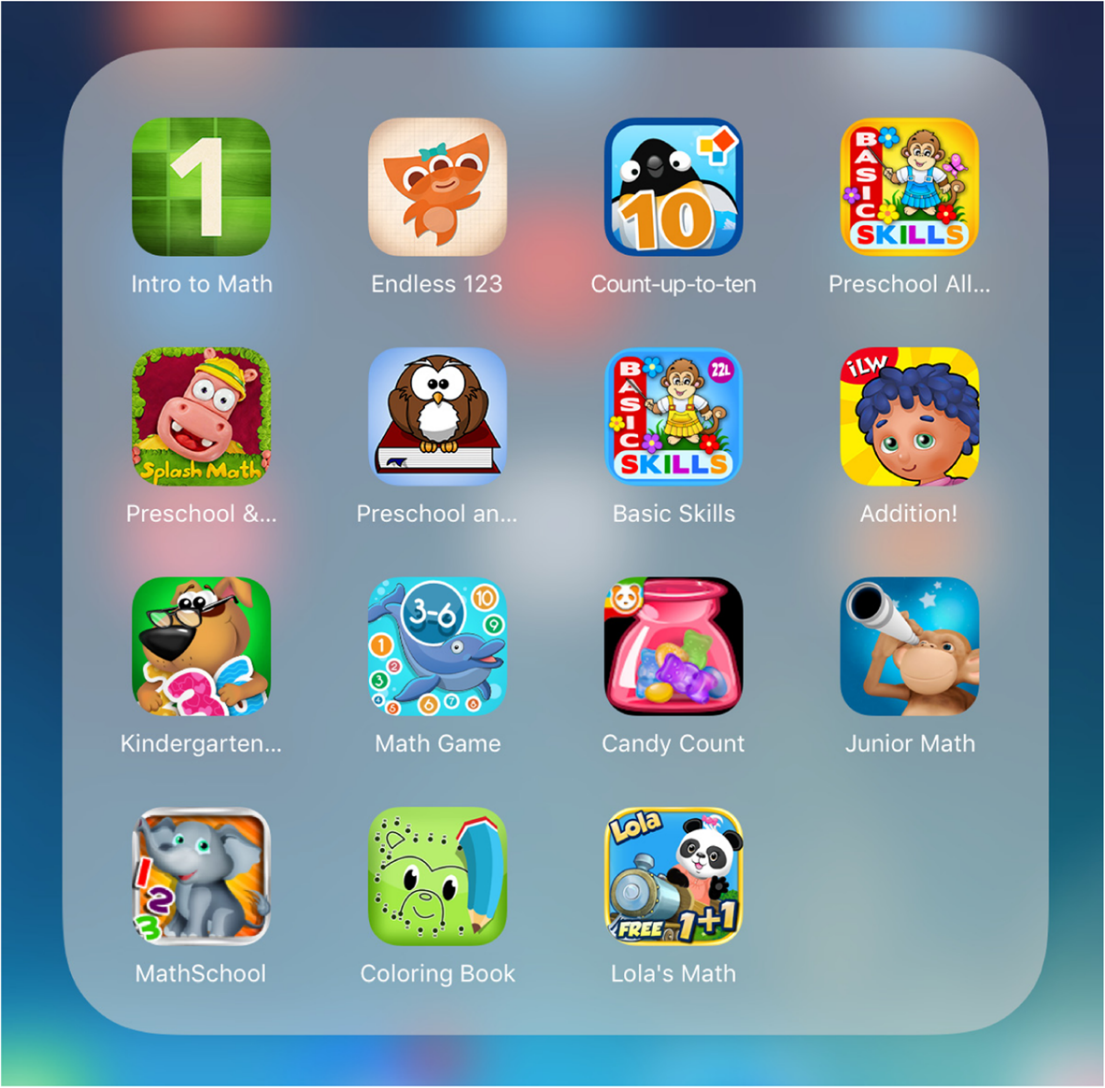
Apps used in study

The intervention began using 10 apps for the first week and then a new app was introduced each day thereafter for a total of 15 apps. Children would receive instruction on how to use a particular app at the start of the lesson followed by time to play with the app. In the second part of the lesson, children could choose whatever app they preferred for the remainder of the lesson. The apps were downloaded from the Apple Store for free or for a nominal fee. The app icons used in this study are shown in Fig. 1.

Table 1, below, summarizes the types of skill development in each of the apps.

**Table 1:** Apps and skills

App	Numeracy skills
Intro to Math	Number drawing, sorting numbers, subitizing, sorting objects
Endless 123	Number recognition, number sorting, addition with numerals objects, number drawing
Count-up-to-ten	Counting, associating objects with a numeral, number drawing,
Preschool All-in-one	Counting, patterns
Pre-school & Kindergarten Splash Math	Identify missing number in an equation (e.g., 10 = ? + 8)
Preschool & Kindergarten Learning Games	Counting, addition, subtraction
Basic Skills	Counting, patterns
Addition	Add up to 6, add up to 10, add up to 15
Kindergarten	Addition
Math Game	Counting, addition, subtraction
Candy Count	Counting, subitizing, comparing quantities (more, less, the same), sort colors
Junior Math	Counting, patterns
Math School	Number drawing, addition, subtraction, counting (dot to dot)
Coloring Book	Counting (dot to dot)
Lola’s Math	Number recognition, more, less, same, sorting

The items used to measure children’s mathematics ability in the pre- and post-tests were created based on the concepts taught in the classroom, which were aligned with the curriculum outcomes as previously noted. A map of the curriculum outcome and corresponding items is shown in Table 4 in the Appendix. These items were modeled from the exemplars provided in the provincial curriculum document and in consultation with the teacher (Department of Education, Early Learning and Culture of PE 2008).

### Data collection

**Fig. 2:**
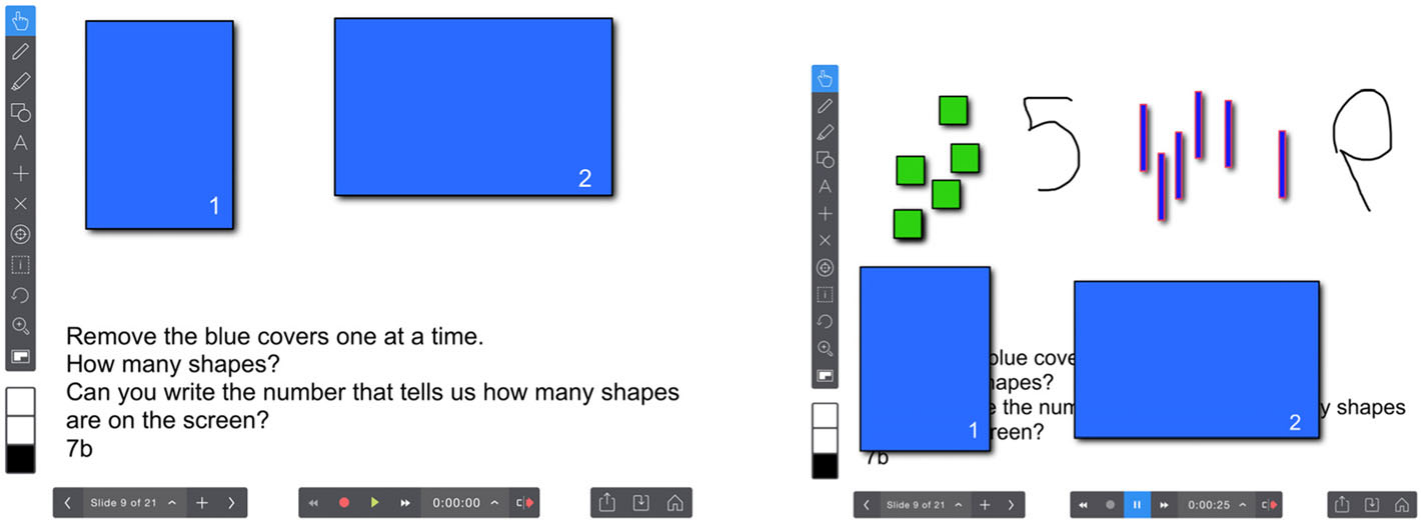
Original test item and test item after answered by student

Data in the form of children’s numeracy test scores was collected using an application called Explain Everything. This application is an interactive screen-casting whiteboard, which stored the test items and recorded children’s responses to each item. To capture children’s responses, the examiner would orally read instructions that were printed on the bottom of each test page and then the child would respond by speaking, writing, or manipulating objects on the screen; all of which were captured using Explain Everything simultaneous video and audio recording feature known as screen casting. Figure 2 shows an original test item as presented in Explain Everything and how a student manipulated the objects on the screen (on the right) to demonstrate their understanding.

The researcher and a research assistant independently scored each child’s test by reviewing each screen-cast. When the scores did not match, we discussed our responses and agreed on a score. Each item was scored based on a 4-point rubric: (1) Do not know or responded incorrectly, (2) demonstrated some understanding of the concept but response was not correct, (3) provided a correct response but the strategy was not strategic or efficient, and (4) provided a correct response that was efficient. An example of a level 2 response would be a number written backwards or upside down and an example of a level 3 response is writing the number seven starting from the bottom and moving to the top (i.e., drawing the number from the finish position to the start position). The kindergarten teacher reviewed and agreed with the 4-point rubric. In the sample response shown in Fig. 2, the child would receive a 4 for the first answer (i.e., 5) but a 3 for the second answer because the child orally indicated the set containing six elements but they had difficulty writing the digit 6.

The pre-test at time 1 and post-test at time 2 contained the same items except that the colors or the shape of objects were changed. Prior to implementing the study, the test was piloted with three children from another school and reviewed by the kindergarten teacher. Small changes to the printed instructions on each slide were made to better align the vocabulary with children’s understanding.

During the experimental phase, the research assistants recorded field notes documenting children’s behaviors and the apps that were most favored. Children’s feedback on what they liked about the apps (i.e., the characteristics of the apps) and other observations were also documented.

### Analysis

Mean scores were calculated for both groups on the pre-test. Due to the small sample size, it was not possible to conduct the stringent inferential analysis of covariance; therefore, group difference scores were analyzed. The observational field notes were conceptually analyzed to determine the presence of common words or phrases to make inferences about the observations. The coding began with predefined categories such as collaboration, level of engagement, and choice of apps but was flexible to allow for the addition of other unanticipated themes.

## Results

Cronbach’s alpha was used to measure the internal consistency of the scale. After removing poor performing items due to poor discrimination, 22 items remained. The items that discriminated poorly were due to all students receiving the top score on the item; subsequently, there was no discrimination between ability. Easy items were purposefully included on the assessment to ease students into the testing; hence, it was expected that a number of items would be removed from the test due to poor discrimination. Cronbach’s alpha for the remaining 22-item scale on the pre-test was 0.803 and 0.805 for the post-test. Thus, the coefficient exceeded the absolute minimum threshold of 0.7 but also met the ideal minimum threshold of 0.8 (Tabachnick and Fidell 2012), indicating a reliable set of test items.

### Descriptive summary of items

Group 1 consisted of four females and three males, and group 2 had two females and four males. All children were 4 or 5 years old. Items ranged in difficulty with the hardest being items 1.7b (write the number 6; *M* = 2.46, *SD* = 1.27), 1.7c (write the number 5; *M* = 2.92, *SD* = 1.19), and 1.2h (recognize seven dots on a 10 frame; *M* = 2.92, *SD* = 0.28). Easier items were 1.4b (create a set of 7; *M* = 3.77, *SD* = 0.83), 1.7k (count backwards from 5; *M* = 3.67, *SD* = 0.89), 2.1d (identify repeating and non-repeating patterns; *M* = 3.54, *SD* = 1.13), 1.2a (identify 3 on a die, *M* = 3.54, *SD* = 0.52), and 1.2b (identify 4 on a die, *M* = 3.54, *SD* = 0.52).

Given that four out of 10 apps involved drawing numbers, an increase in this skill was anticipated from the pre- to the post-test. However, of the two items assessing drawing numbers, only one item (i.e., 1.7b drawing the number 6) revealed a significant increase from *M* = 2.86 and *SD* = 1.46 to *M* = 3.29 and *SD* = 1.25, following the intervention.

When comparing mean scores, the experimental and control group differed by 0.01 on the pre-test (see Table 2). After the intervention, the experimental group increased slightly (+ 0.02) and the control group decreased slightly (− 0.04). On the post-test, the two groups differed by 0.05 with the experimental group having the higher mean score (see Table 2). These differences are too small to suggest the intervention had any effect on students’ mathematics ability.

**Table 2:** Pre- and post-test scores x intervention

	Experimental			Control		
	*N*	*M*	*SD*	*N*	*M*	*SD*
Pre-test	7	3.29	0.451	6	3.30	0.358
Post-test	7	3.31	0.442	6	3.26	0.335

### Observational findings

All children were keen to use the iPads over the 10 days of mathematics lessons as they asked the teacher each day when the research team was arriving so that they could use the iPads. During the introduction to a new app phase at the beginning of each guided mathematics lesson, where the children were shown how to use an app (if they needed help), children did not use a headset so that they could hear the instructions (volume was turned down on the ipads). During this guided instruction, children were more apt to collaborate with each other to share what was on their screens and provide help to get to another level or step. When the children wore a headset, there was a greater tendency for children to focus on their own screen as they were not distracted by sounds coming from other iPads or giggles and exclamations coming from their peers.

The four stronger children in the class (as identified by the teacher and confirmed by the pre-test) appeared to have a better understanding of how to maneuver through different levels in an app whereas the weaker children frequently needed guidance on how to proceed to the next level. Another observation related to levels in an app was the difficulty of the level. When an the app level became too challenging, children would either look to abandon the app or randomly select answers until they eliminated all incorrect responses and identified the correct response. An example of this type of question was the equation 2 + 3 = ? which was supplemented by corresponding dots along with responses of 4, 5, and 6. When we debriefed the teacher about this trial and error process of selecting the correct response, she believed that children were learning more than we were giving them credit for which was encouraging; however, we were still concerned that children may be learning by memorizing rather than having a conceptual understanding of the concept. Another finding related to children’s ability was that stronger children exhibited more independence in using apps. These children were able to use a new app by listening to the audio instructions provided in the app or were confident enough to skip over the audio instructions and starting using the app immediately. In comparison, weaker children frequently needed the research assistant to provide oral instruction as well as provide a demonstration (i.e., model using the app) for new apps.

When drawing numbers, three children would opt to use their index finger to trace the number. Given that children were still developing their fine motor skills, we believed that it was important to encourage children to use a stylus; hence, we encouraged children to use a stylus at all times but particularly when they were drawing numbers.

After every 3 days, children were asked, what was their favorite app? The last app that they played was the most common response, likely because it was the most current in their mind. When prompted further to think about the other apps, the most favored apps were not the most pedagogically structured apps but rather apps that had a lot of bling. For example, children were drawn to apps that had exploding stars when they completed a set of tasks or would see a funny character dance across the screen (e.g., Endless 123). In the same vein, children were quick to move from one app to another. For example, all children quickly grew tired of number drawing and when left to their own choices they would not select apps with this skill development. When debriefing this finding with the teacher she noted that an app tended to have a life expectancy of a few days and then children would become tired with the simplicity of the app unless the app captured their attention with bling. Table 3 displays the app and frequency of children’s preference for the app, which was based on the frequency they choose the app during their structured playtime. This is a holistic measure taking into consideration that there were parts of an app children played frequently, while other parts of the app were ignored. The frequency is also influenced by mathematics ability in that more challenging apps were used less frequently by weaker children.

**Table 3:** Frequency of use

App	1	2	3
Intro to Math	✔		
Endless 123			✔
Count-up-to-ten			✔
Preschool All-in-one		✔	
Splash Math, Preschool & Kindergarten	✔		
Preschool & Kindergarten Learning Games		✔	
Basic Skills	✔		
Addition		✔	
Kindergarten		✔	
Math Game		✔	
Candy Count			✔
Junior Math		✔	
Math School		✔	
Coloring Book	✔		
Lola’s Math		✔	

Note: 1 = least frequent, 2 = moderate use, 3 = daily use

In terms of the teacher’s experience with the iPads, pre-experiment meetings revealed that she was completely new to using iPads and did not own an iPad of her own. She received instruction on how to (a) access the internet, (b) link the iPads so that apps could be downloaded automatically to more than one device, (c) download apps, (d) organize apps into a folder, and (e) delete apps that were no longer being used or accidentally downloaded. Throughout the study, the teacher would periodically sought assistance for these tasks. Following the study, we met twice to solve problems related to purchasing new apps and simultaneously downloading them to all four devices.

## Discussion

This discussion is framed by the two research questions posed in this study. In addition, other insights garnered in the study are discussed.

### To what extent does the use of mathematical apps using iPads enhance children’s learning of numeracy in kindergarten?

Considering the small gains in achievement by the experimental group in comparison to a slight decrease in achievement in the control group, there was no significant difference in children’s understanding of numeracy as measured on the pre- and post-tests between the two groups. Although a difference was anticipated based on prior research, these findings were the same as Mattoon et al. (2015) who also reported small gains but no significant difference between their two groups in a 6-week long study. Despite the absence of significant gains between the two groups, this study provides evidence that using technology in this context did not deter or lessen children’s development of numeracy skills. This study adds to the work of Bebell et al. (2012) who reported that iPads do not hinder early learning of literacy. We can now conclude that iPads do not hinder early learning of numeracy as well as literacy. This is an important finding that will broaden the utility of iPads in the early years classroom. In summary, the use of mathematical apps on iPads slightly enhanced children’s learning of mathematics as shown by gains from the pre- to the post-test; however, the gains were not significant between groups.

### What factors influence children’s use of interactive technology in a play-based learning environment?

Factors that influenced children’s use of interactive technology focused on (a) collaboration, (b) ability, (c) use of a stylus, (d) maturity, and (e) teachers’ skill level. A factor known to influence children’s use of interactive technology was their affinity for collaboration. Given that prior research documented how technology fostered a collaborative learning environment (Shifflet et al. 2012), it was not surprising that children naturally collaborated without any guidance to do so from the researcher. During the introduction phase of a new app, children naturally gravitated towards each other to share what was on their iPad as well as to help each other progress to the next level or game. This affinity for collaboration is an asset to learning mathematics that needs to be supported so that when children leave the play-based learning environment they are still drawn to helping each other with tasks in general but specifically, in the learning of mathematics.

Another observation focused on the impact of children’s prior mathematics ability and experience with apps. No research was found that examined the relationship between children’s mathematics ability and interaction with apps. However, Hung et al. (2015) reported that when children were challenged, they reported higher levels of engagement and satisfaction. Extending Hung et al.’s (2015) finding, it appears that children with stronger skills in mathematics were more apt to persevere and be engaged with the app. In contrast, weaker children were more apt to abandon the app or use a trial and error process to progress to the next level, in which case, the quality of their learning was questioned, as learning may be memorized rather than conceptual. Children’s ability level can be connected to attention span since apps that required greater concentration would be met with a shorter attention span. This short attention span appeared to be age appropriate considering the attention span for 4- to 5-year-olds is a maximum 6 to 7 min (i.e., chronological age + 1); although attention span for children playing or being socially engaged can exceed, these maximum times are typically reserved for formal instruction (Wesson 2011). Hence, challenging and less creative apps (e.g., Montessori Math) might be perceived more like a formal lesson whereas entertaining and creative apps with bling (e.g., Count-up-to-ten) may be perceived as play.

Further endorsing the need for more research in this area was the absence of research focusing on the use of a stylus versus the index finger to interact with iPads. Although children naturally gravitated towards using their index finger, we encouraged children to use the stylus to reinforce printing skills learned with a pencil. More research is needed to corroborate this practice.

The fact that children in this study gravitated towards apps that stimulated laughter through humorous animated characters or bursts of stars or sparkles confirmed our thoughts about their level of maturity and corresponding desire for play. Once children had been exposed to the new app each day, they were able to choose any app to play with for the remaining time. The outcome of children’s decision-making resulted in the selection of apps that were creative and fun in contrast to other apps that were more pedagogically accurate containing appropriate levels of difficulty and sequencing of questions. This finding needs further research to explore the extent to which this affinity for creative and fun apps continues through the elementary and primary grades.

The last factor that influenced children’s use of interactive technology was the teachers’ skill level and interest in implementing apps as curricular learning resources. As noted above, the teacher participating in this study could be described as a beginner in using interactive technology and her skill set was similar to what was previous documented as limited to downloading images for presentations (Public Broadcasting Service and Grunwald Associates 2009, 2011). However, her interest, rather than ability, was the catalyst for implementing technology in her classroom and through opportunities for professional development as called for by other researchers (i.e., Simon et al. 2013); this teacher can now be described as experienced and innovative in her use of interactive technology in the classroom. Key to this transformation was providing professional development in a one-to-one session and in an as-needed basis.

## Conclusions

With the advancement of interactive technology and more user-friendly touchable interfaces, the use of these devices in early years classrooms is not only suitable but also appropriate in preparing early years children for the technological world they will live in (Gordon and Williams Browne 2016). This study revealed that children using interactive technology in the form of mathematics apps as part of a play-based learning environment for mathematics had small gains in achievement as measured using a pre- and post-test. Although the gains in achievement were not significant between the control and intervention groups, learning using interactive technology did not lessen children’s opportunity to learn about numeracy concepts as children were observed collaborating and were highly engaged, particularly with apps that were creative. A longer experiment with a larger sample is needed to validate this finding.

Another important finding related to using interactive technology in a play-based learning environment was the need for guided direction in selecting quality apps that supported learning. When children were left to their own accord, they almost always selected apps or segments of apps that were high in entertainment value and low in educational value. Given the premise of play-based learning environments where play is situated in intentional and specific learning contexts that nurtures learning while providing children with independence to choose activities, it is important to select mathematical apps that are not only aligned with the curriculum but are also creative and fun and provide opportunities to learn. In these learning environments, pleasurable, entertaining play is encouraged in balance with other play-based activities that foster learning in specific domains such as numeracy. This finding was connected to children’s attention span which appeared to be dependent on the creativity of the app as well as the difficulty level of the app such that more creative apps held children’s attention spans longer but also the difficulty level influenced student engagement with apps. This finding corroborates the work of Couse and Chen (2010) who reported that engagement increased with age and by extension mathematics ability.

In sum, interactive technology in the form of iPads with mathematical apps promoted student collaboration and engagement. However there is still much to learn about the quality of apps and their impact on children’s learning, particularly when children are encouraged to make choices in a play-based learning environment.

## Appendix

**Table 4:** Curriculum outcome and corresponding items

Outcome	Item
1.1 Count in a variety of ways	1.1a Count backwards from 10 1.1b Count backwards from 5
1.2 Explore a variety of physical representations of numbers 1 to 10	1.2a Identify 3 on a die without counting 1.2b Identify 4 on a die without counting 1.2c Identify 5 on a die without counting 1.2e Recognize 5 dots on a 5 frame 1.2f Recognize 4 dots on a 5 frame 1.2g Recognize 10 dots on a 10 frame 1.2h Recognize 7 dots on a 10 frame
1.3 Count to determine the number in a group (0 to 10)	1.3a Count to determine a group of 10
1.4 Create sets of a given number (0 to 10)	1.4a Create a set of 3 1.4b Create a set of 7
1.5 Show a given number as two parts concretely and name the two parts (2 to 10)	1.5b Use adding-on to add 3 orange circles plus 5 orange circles.
1.6 Determine which group has more, which group has less, or which are equivalent	1.6c Which group has more? less? or the same?
1.7 Use symbols to represent numbers in a variety of meaningful contexts	1.7j Count backwards from 10 1.7k Count backwards from 5 1.7c Write the number 5
2.1 Demonstrate an understanding of repeating patterns (two or three elements) by identifying, describing, copying, extending, and creating patterns	2.1c Repeating complex patter: ABCCABCC 2.1d Identify repeating and non-repeating pattern 2.1b Complex AA/B/AA/B 2.1b 3 elements: Complex A/B/CC/A/B/CC
3.1 Compare two objects based on a single attribute such as (height) and volume (capacity)	3.1a Of the 5 bars, which is the tallest bar? 3.1b Of the 5 bars, which is the shortest bar? 3.1c Put the bars in order of short to tall. 3.1d Put the balloons in order of big to small (complex)
